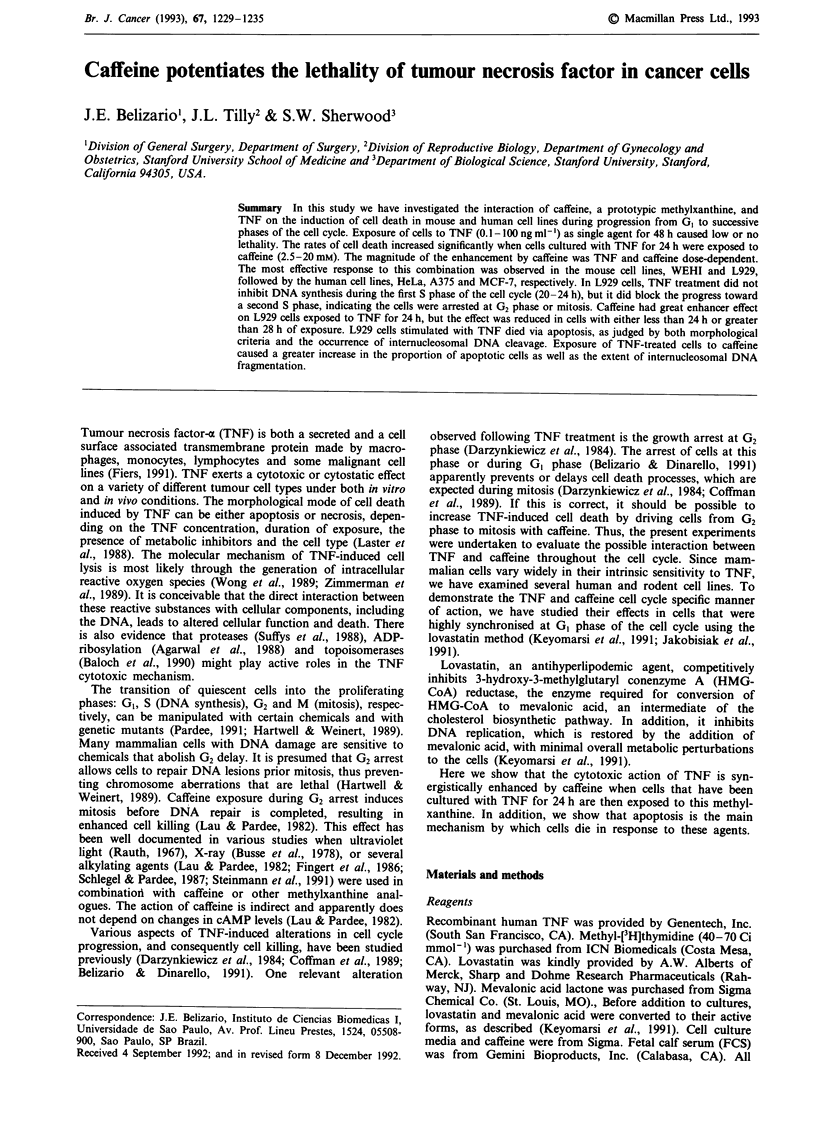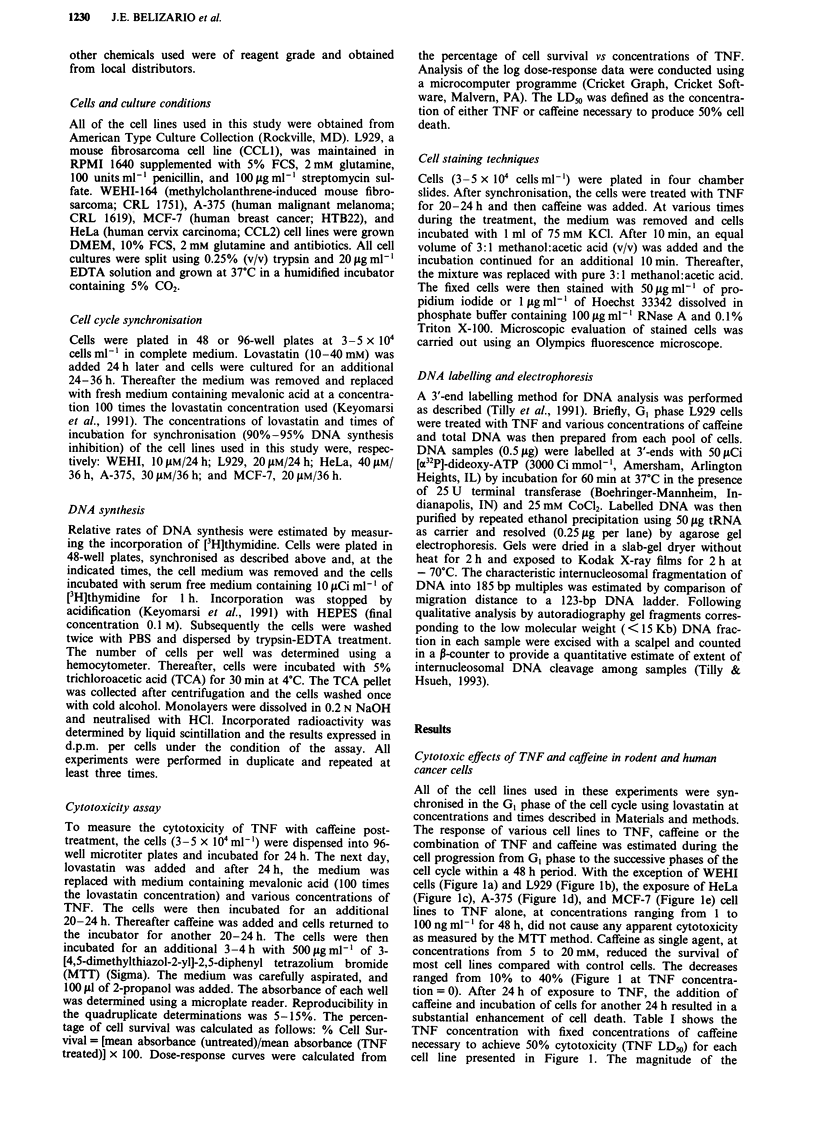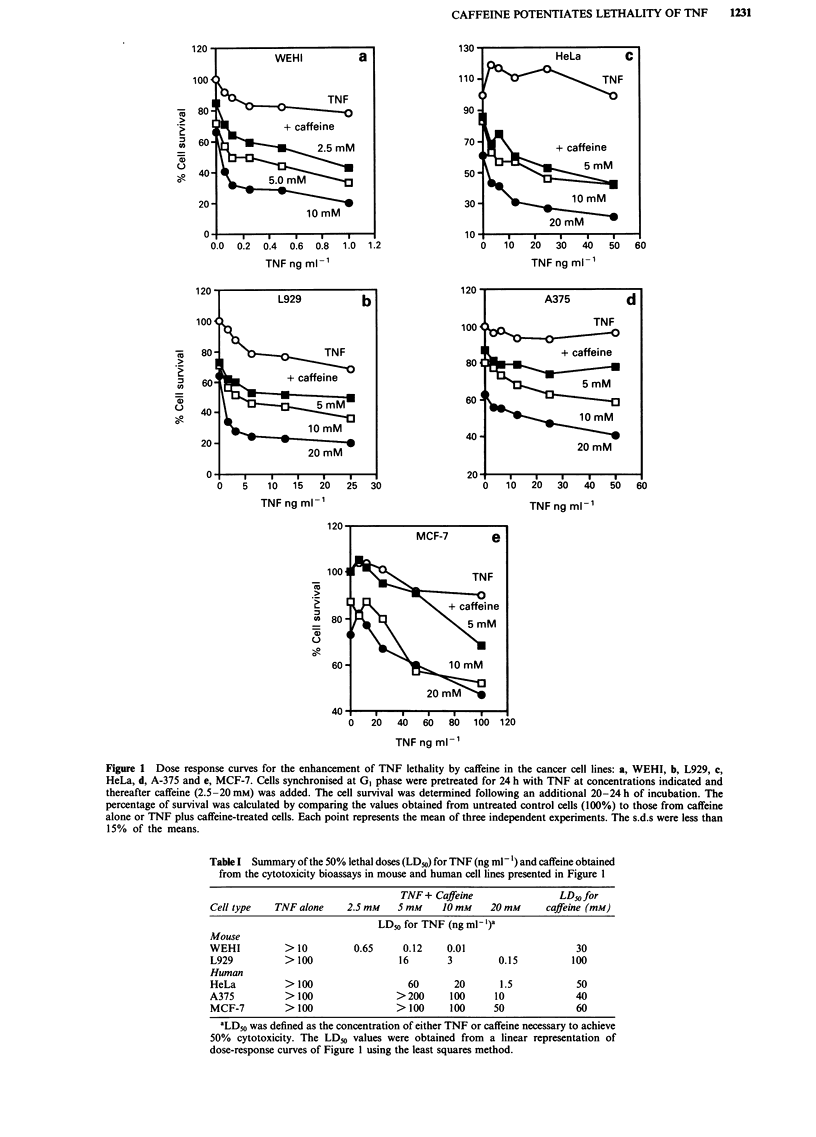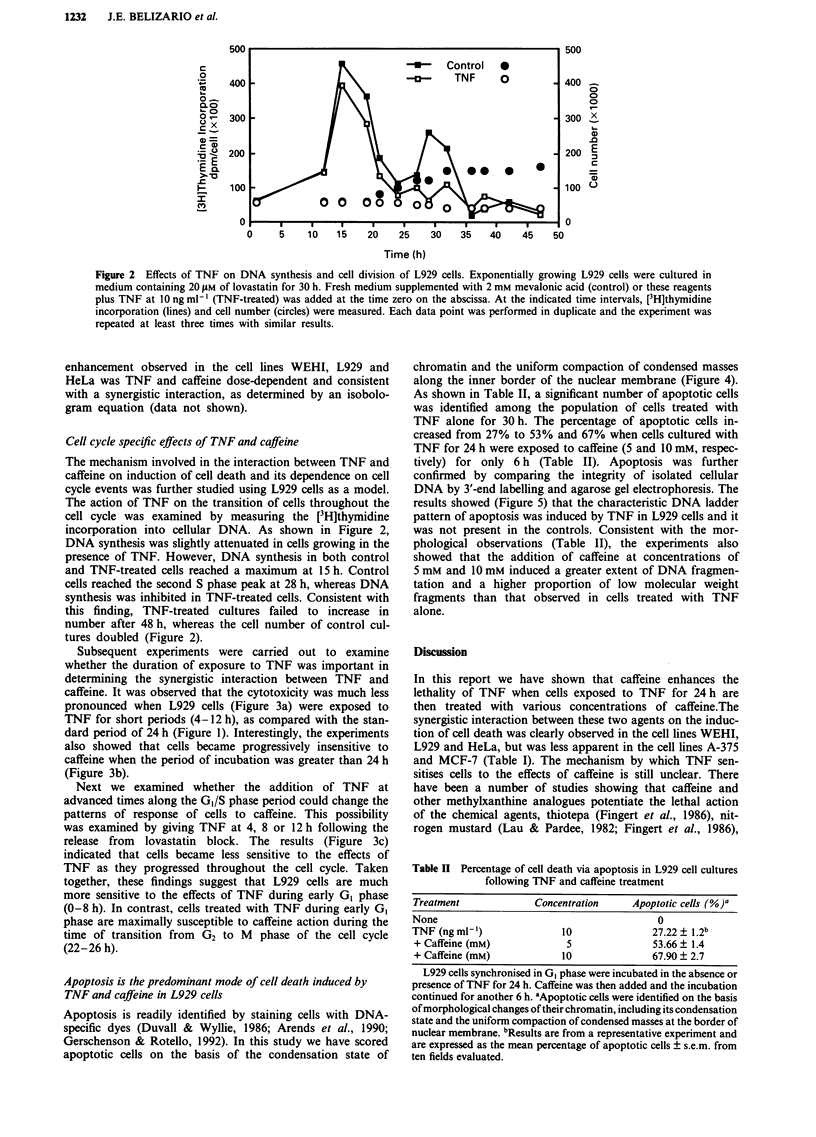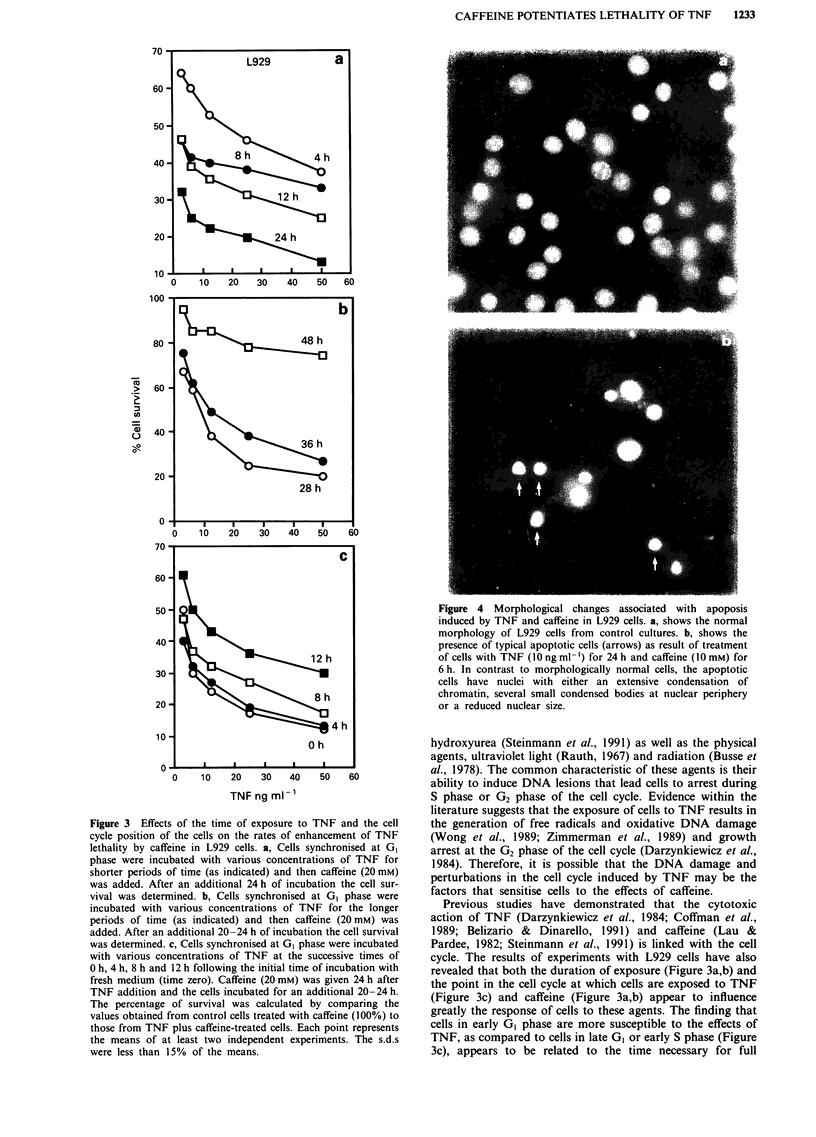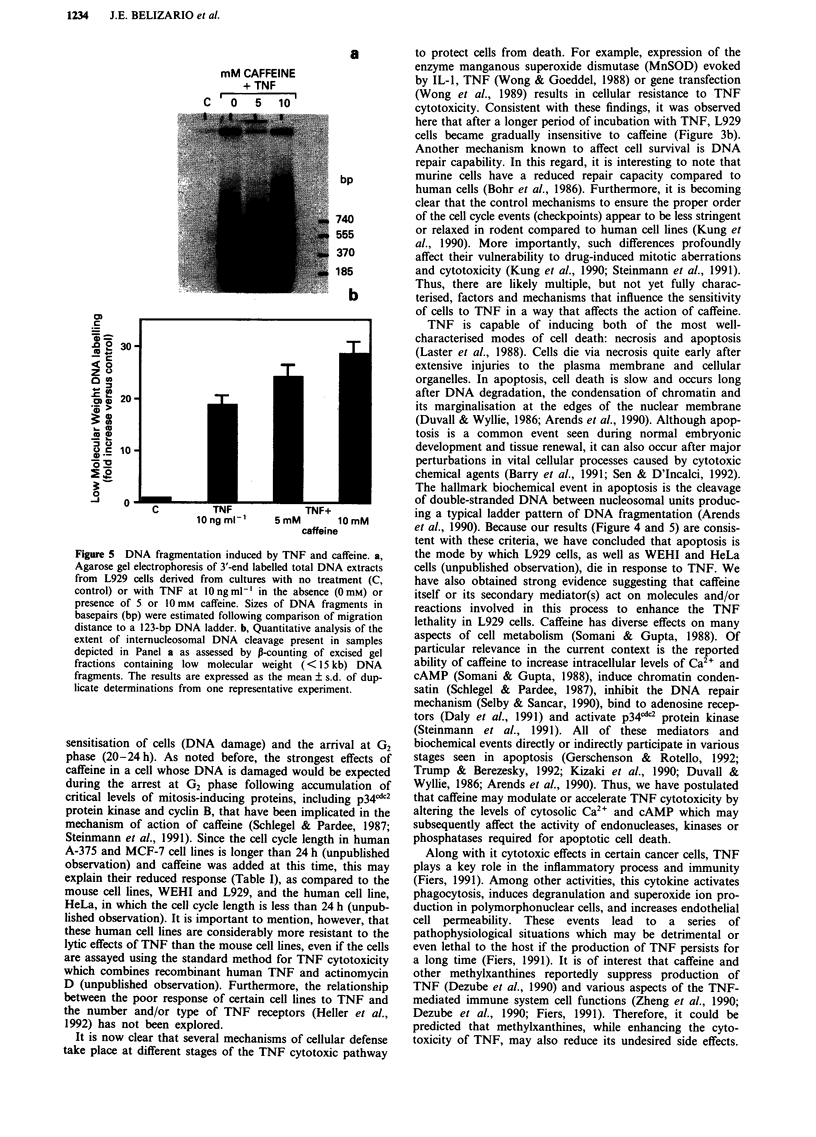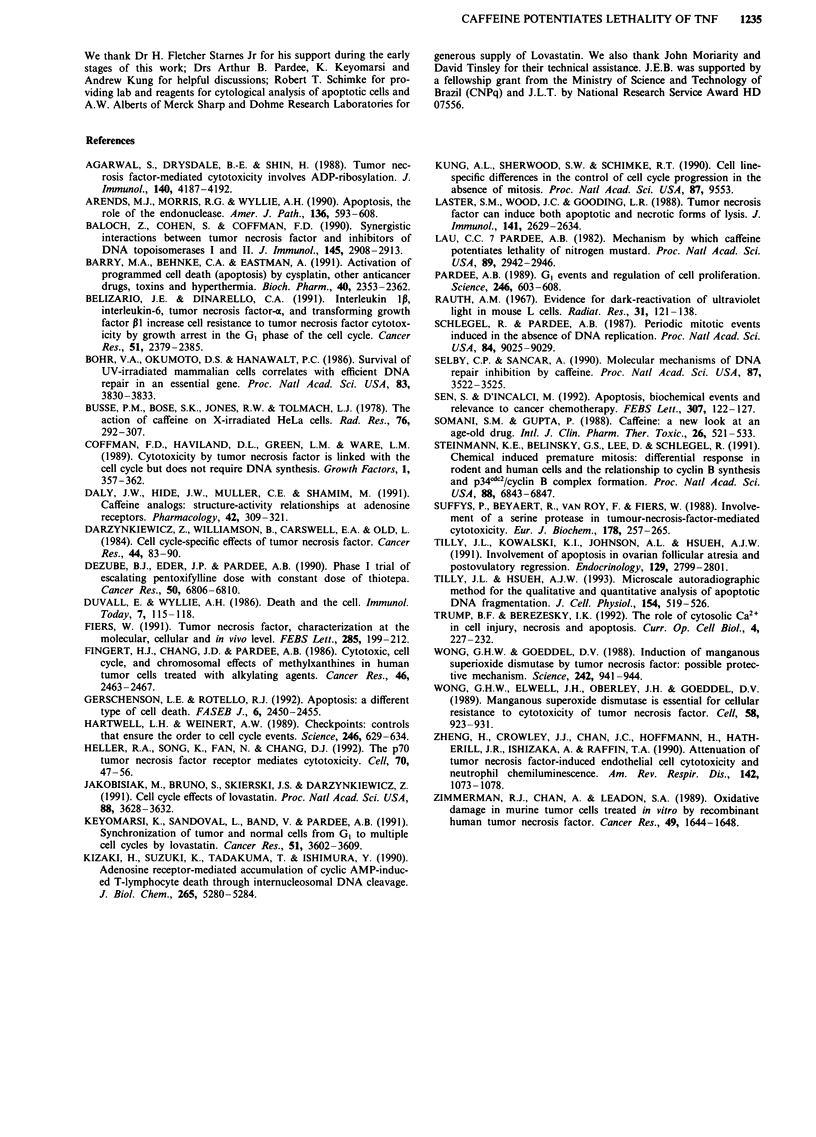# Caffeine potentiates the lethality of tumour necrosis factor in cancer cells.

**DOI:** 10.1038/bjc.1993.230

**Published:** 1993-06

**Authors:** J. E. Belizario, J. L. Tilly, S. W. Sherwood

**Affiliations:** Department of Surgery, Stanford University School of Medicine, California 94305.

## Abstract

**Images:**


Br. J. Cancer (1993), 67, 1229–1235 © Macmillan Press Ltd., 1993

### Caffeine potentiates the lethality of tumour necrosis factor in cancer cells

J.E. Belizario[1], J.L. Tilly[2] & S.W. Sherwood[3]

[1]*Division of General Surgery, Department of Surgery,* [2]*Division of Reproductive Biology, Department of Gynecology and Obstetrics, Stanford University School of Medicine and* [3]*Department of Biological Science, Stanford University, Stanford, California 94305, USA.*

**Summary** In this study we have investigated the interaction of caffeine, a prototypic methylxanthine, and TNF on the induction of cell death in mouse and human cell lines during progression from $G_1$ to successive phases of the cell cycle. Exposure of cells to TNF (0.1–100 ng $ml^{-1}$) as single agent for 48 h caused low or no lethality. The rates of cell death increased significantly when cells cultured with TNF for 24 h were exposed to caffeine (2.5–20 mM). The magnitude of the enhancement by caffeine was TNF and caffeine dose-dependent. The most effective response to this combination was observed in the mouse cell lines, WEHI and L929, followed by the human cell lines, HeLa, A375 and MCF-7, respectively. In L929 cells, TNF treatment did not inhibit DNA synthesis during the first S phase of the cell cycle (20–24 h), but it did block the progress toward a second S phase, indicating the cells were arrested at $G_2$ phase or mitosis. Caffeine had great enhancer effect on L929 cells exposed to TNF for 24 h, but the effect was reduced in cells with either less than 24 h or greater than 28 h of exposure. L929 cells stimulated with TNF died via apoptosis, as judged by both morphological criteria and the occurrence of internucleosomal DNA cleavage. Exposure of TNF-treated cells to caffeine caused a greater increase in the proportion of apoptotic cells as well as the extent of internucleosomal DNA fragmentation.

Tumour necrosis factor-α (TNF) is both a secreted and a cell surface associated transmembrane protein made by macrophages, monocytes, lymphocytes and some malignant cell lines (Fiers, 1991). TNF exerts a cytotoxic or cytostatic effect on a variety of different tumour cell types under both *in vitro* and *in vivo* conditions. The morphological mode of cell death induced by TNF can be either apoptosis or necrosis, depending on the TNF concentration, duration of exposure, the presence of metabolic inhibitors and the cell type (Laster *et al.*, 1988). The molecular mechanism of TNF-induced cell lysis is most likely through the generation of intracellular reactive oxygen species (Wong *et al.*, 1989; Zimmerman *et al.*, 1989). It is conceivable that the direct interaction between these reactive substances with cellular components, including the DNA, leads to altered cellular function and death. There is also evidence that proteases (Suffys *et al.*, 1988), ADP-ribosylation (Agarwal *et al.*, 1988) and topoisomerases (Baloch *et al.*, 1990) might play active roles in the TNF cytotoxic mechanism.

The transition of quiescent cells into the proliferating phases: $G_1$, S (DNA synthesis), $G_2$ and M (mitosis), respectively, can be manipulated with certain chemicals and with genetic mutants (Pardee, 1991; Hartwell & Weinert, 1989). Many mammalian cells with DNA damage are sensitive to chemicals that abolish $G_2$ delay. It is presumed that $G_2$ arrest allows cells to repair DNA lesions prior mitosis, thus preventing chromosome aberrations that are lethal (Hartwell & Weinert, 1989). Caffeine exposure during $G_2$ arrest induces mitosis before DNA repair is completed, resulting in enhanced cell killing (Lau & Pardee, 1982). This effect has been well documented in various studies when ultraviolet light (Rauth, 1967), X-ray (Busse *et al.*, 1978), or several alkylating agents (Lau & Pardee, 1982; Fingert *et al.*, 1986; Schlegel & Pardee, 1987; Steinmann *et al.*, 1991) were used in combination with caffeine or other methylxanthine analogues. The action of caffeine is indirect and apparently does not depend on changes in cAMP levels (Lau & Pardee, 1982).

Various aspects of TNF-induced alterations in cell cycle progression, and consequently cell killing, have been studied previously (Darzynkiewicz *et al.*, 1984; Coffman *et al.*, 1989; Belizario & Dinarello, 1991). One relevant alteration observed following TNF treatment is the growth arrest at $G_2$ phase (Darzynkiewicz *et al.*, 1984). The arrest of cells at this phase or during $G_1$ phase (Belizario & Dinarello, 1991) apparently prevents or delays cell death processes, which are expected during mitosis (Darzynkiewicz *et al.*, 1984; Coffman *et al.*, 1989). If this is correct, it should be possible to increase TNF-induced cell death by driving cells from $G_2$ phase to mitosis with caffeine. Thus, the present experiments were undertaken to evaluate the possible interaction between TNF and caffeine throughout the cell cycle. Since mammalian cells vary widely in their intrinsic sensitivity to TNF, we have examined several human and rodent cell lines. To demonstrate the TNF and caffeine cell cycle specific manner of action, we have studied their effects in cells that were highly synchronised at $G_1$ phase of the cell cycle using the lovastatin method (Keyomarsi *et al.*, 1991; Jakobisiak *et al.*, 1991).

Lovastatin, an antihyperlipodemic agent, competitively inhibits 3-hydroxy-3-methylglutaryl conenzyme A (HMG-CoA) reductase, the enzyme required for conversion of HMG-CoA to mevalonic acid, an intermediate of the cholesterol biosynthetic pathway. In addition, it inhibits DNA replication, which is restored by the addition of mevalonic acid, with minimal overall metabolic perturbations to the cells (Keyomarsi *et al.*, 1991).

Here we show that the cytotoxic action of TNF is synergistically enhanced by caffeine when cells that have been cultured with TNF for 24 h are then exposed to this methylxanthine. In addition, we show that apoptosis is the main mechanism by which cells die in response to these agents.

### Materials and methods

### *Reagents*

Recombinant human TNF was provided by Genentech, Inc. (South San Francisco, CA). Methyl-[$^3$H]thymidine (40–70 Ci $mmol^{-1}$) was purchased from ICN Biomedicals (Costa Mesa, CA). Lovastatin was kindly provided by A.W. Alberts of Merck, Sharp and Dohme Research Pharmaceuticals (Rahway, NJ). Mevalonic acid lactone was purchased from Sigma Chemical Co. (St. Louis, MO)., Before addition to cultures, lovastatin and mevalonic acid were converted to their active forms, as described (Keyomarsi *et al.*, 1991). Cell culture media and caffeine were from Sigma. Fetal calf serum (FCS) was from Gemini Bioproducts, Inc. (Calabasa, CA). All

Correspondence: J.E. Belizario, Instituto de Ciencias Biomedicas I, Universidade de Sao Paulo, Av. Prof. Lineu Prestes, 1524, 05508-900, Sao Paulo, SP Brazil.
Received 4 September 1992; and in revised form 8 December 1992.

other chemicals used were of reagent grade and obtained from local distributors.

*Cells and culture conditions*

All of the cell lines used in this study were obtained from American Type Culture Collection (Rockville, MD). L929, a mouse fibrosarcoma cell line (CCL1), was maintained in RPMI 1640 supplemented with 5% FCS, 2 mM glutamine, 100 units $ml^{-1}$ penicillin, and 100 $\mu g\,ml^{-1}$ streptomycin sulfate. WEHI-164 (methylcholanthrene-induced mouse fibrosarcoma; CRL 1751), A-375 (human malignant melanoma; CRL 1619), MCF-7 (human breast cancer; HTB22), and HeLa (human cervix carcinoma; CCL2) cell lines were grown DMEM, 10% FCS, 2 mM glutamine and antibiotics. All cell cultures were split using 0.25% (v/v) trypsin and 20 $\mu g\,ml^{-1}$ EDTA solution and grown at 37°C in a humidified incubator containing 5% $CO_2$.

*Cell cycle synchronisation*

Cells were plated in 48 or 96-well plates at $3-5 \times 10^4$ cells $ml^{-1}$ in complete medium. Lovastatin (10–40 mM) was added 24 h later and cells were cultured for an additional 24–36 h. Thereafter the medium was removed and replaced with fresh medium containing mevalonic acid at a concentration 100 times the lovastatin concentration used (Keyomarsi *et al.*, 1991). The concentrations of lovastatin and times of incubation for synchronisation (90%–95% DNA synthesis inhibition) of the cell lines used in this study were, respectively: WEHI, 10 $\mu$M/24 h; L929, 20 $\mu$M/24 h; HeLa, 40 $\mu$M/36 h, A-375, 30 $\mu$M/36 h; and MCF-7, 20 $\mu$M/36 h.

*DNA synthesis*

Relative rates of DNA synthesis were estimated by measuring the incorporation of [$^3$H]thymidine. Cells were plated in 48-well plates, synchronised as described above and, at the indicated times, the cell medium was removed and the cells incubated with serum free medium containing 10 $\mu Ci\,ml^{-1}$ of [$^3$H]thymidine for 1 h. Incorporation was stopped by acidification (Keyomarsi *et al.*, 1991) with HEPES (final concentration 0.1 M). Subsequently the cells were washed twice with PBS and dispersed by trypsin-EDTA treatment. The number of cells per well was determined using a hemocytometer. Thereafter, cells were incubated with 5% trichloroacetic acid (TCA) for 30 min at 4°C. The TCA pellet was collected after centrifugation and the cells washed once with cold alcohol. Monolayers were dissolved in 0.2 N NaOH and neutralised with HCl. Incorporated radioactivity was determined by liquid scintillation and the results expressed in d.p.m. per cells under the condition of the assay. All experiments were performed in duplicate and repeated at least three times.

*Cytotoxicity assay*

To measure the cytotoxicity of TNF with caffeine post-treatment, the cells ($3-5 \times 10^4\,ml^{-1}$) were dispensed into 96-well microtiter plates and incubated for 24 h. The next day, lovastatin was added and after 24 h, the medium was replaced with medium containing mevalonic acid (100 times the lovastatin concentration) and various concentrations of TNF. The cells were then incubated for an additional 20–24 h. Thereafter caffeine was added and cells returned to the incubator for another 20–24 h. The cells were then incubated for an additional 3–4 h with 500 $\mu g\,ml^{-1}$ of 3-[4,5-dimethylthiazol-2-yl]-2,5-diphenyl tetrazolium bromide (MTT) (Sigma). The medium was carefully aspirated, and 100 $\mu$l of 2-propanol was added. The absorbance of each well was determined using a microplate reader. Reproducibility in the quadruplicate determinations was 5–15%. The percentage of cell survival was calculated as follows: % Cell Survival = [mean absorbance (untreated)/mean absorbance (TNF treated)] × 100. Dose-response curves were calculated from the percentage of cell survival *vs* concentrations of TNF. Analysis of the log dose-response data were conducted using a microcomputer programme (Cricket Graph, Cricket Software, Malvern, PA). The $LD_{50}$ was defined as the concentration of either TNF or caffeine necessary to produce 50% cell death.

*Cell staining techniques*

Cells ($3-5 \times 10^4$ cells $ml^{-1}$) were plated in four chamber slides. After synchronisation, the cells were treated with TNF for 20–24 h and then caffeine was added. At various times during the treatment, the medium was removed and cells incubated with 1 ml of 75 mM KCl. After 10 min, an equal volume of 3:1 methanol:acetic acid (v/v) was added and the incubation continued for an additional 10 min. Thereafter, the mixture was replaced with pure 3:1 methanol:acetic acid. The fixed cells were then stained with 50 $\mu g\,ml^{-1}$ of propidium iodide or 1 $\mu g\,ml^{-1}$ of Hoechst 33342 dissolved in phosphate buffer containing 100 $\mu g\,ml^{-1}$ RNase A and 0.1% Triton X-100. Microscopic evaluation of stained cells was carried out using an Olympics fluorescence microscope.

*DNA labelling and electrophoresis*

A 3′-end labelling method for DNA analysis was performed as described (Tilly *et al.*, 1991). Briefly, $G_1$ phase L929 cells were treated with TNF and various concentrations of caffeine and total DNA was then prepared from each pool of cells. DNA samples (0.5 $\mu$g) were labelled at 3′-ends with 50 $\mu$Ci [$\alpha^{32}$P]-dideoxy-ATP (3000 Ci $mmol^{-1}$, Amersham, Arlington Heights, IL) by incubation for 60 min at 37°C in the presence of 25 U terminal transferase (Boehringer-Mannheim, Indianapolis, IN) and 25 mM $CoCl_2$. Labelled DNA was then purified by repeated ethanol precipitation using 50 $\mu$g tRNA as carrier and resolved (0.25 $\mu$g per lane) by agarose gel electrophoresis. Gels were dried in a slab-gel dryer without heat for 2 h and exposed to Kodak X-ray films for 2 h at − 70°C. The characteristic internucleosomal fragmentation of DNA into 185 bp multiples was estimated by comparison of migration distance to a 123-bp DNA ladder. Following qualitative analysis by autoradiography gel fragments corresponding to the low molecular weight (< 15 Kb) DNA fraction in each sample were excised with a scalpel and counted in a $\beta$-counter to provide a quantitative estimate of extent of internucleosomal DNA cleavage among samples (Tilly & Hsueh, 1993).

### Results

*Cytotoxic effects of TNF and caffeine in rodent and human cancer cells*

All of the cell lines used in these experiments were synchronised in the $G_1$ phase of the cell cycle using lovastatin at concentrations and times described in Materials and methods. The response of various cell lines to TNF, caffeine or the combination of TNF and caffeine was estimated during the cell progression from $G_1$ phase to the successive phases of the cell cycle within a 48 h period. With the exception of WEHI cells (Figure 1a) and L929 (Figure 1b), the exposure of HeLa (Figure 1c), A-375 (Figure 1d), and MCF-7 (Figure 1e) cell lines to TNF alone, at concentrations ranging from 1 to 100 ng $ml^{-1}$ for 48 h, did not cause any apparent cytotoxicity as measured by the MTT method. Caffeine as single agent, at concentrations from 5 to 20 mM, reduced the survival of most cell lines compared with control cells. The decreases ranged from 10% to 40% (Figure 1 at TNF concentration = 0). After 24 h of exposure to TNF, the addition of caffeine and incubation of cells for another 24 h resulted in a substantial enhancement of cell death. Table I shows the TNF concentration with fixed concentrations of caffeine necessary to achieve 50% cytotoxicity (TNF $LD_{50}$) for each cell line presented in Figure 1. The magnitude of the

**Figure 1** Dose response curves for the enhancement of TNF lethality by caffeine in the cancer cell lines: **a**, WEHI, **b**, L929, **c**, HeLa, **d**, A-375 and **e**, MCF-7. Cells synchronised at $G_1$ phase were pretreated for 24 h with TNF at concentrations indicated and thereafter caffeine (2.5–20 mM) was added. The cell survival was determined following an additional 20–24 h of incubation. The percentage of survival was calculated by comparing the values obtained from untreated control cells (100%) to those from caffeine alone or TNF plus caffeine-treated cells. Each point represents the mean of three independent experiments. The s.d.s were less than 15% of the means.

**Table I** Summary of the 50% lethal doses ($LD_{50}$) for TNF (ng $ml^{-1}$) and caffeine obtained from the cytotoxicity bioassays in mouse and human cell lines presented in Figure 1

| *Cell type* | *TNF alone* | *TNF + Caffeine* 2.5 mM | 5 mM | 10 mM | 20 mM | *$LD_{50}$ for caffeine (mM)* |
|---|---|---|---|---|---|---|
| | | $LD_{50}$ for TNF (ng $ml^{-1}$)[a] | | | | |
| *Mouse* | | | | | | |
| WEHI | >10 | 0.65 | 0.12 | 0.01 | | 30 |
| L929 | >100 | | 16 | 3 | 0.15 | 100 |
| *Human* | | | | | | |
| HeLa | >100 | | 60 | 20 | 1.5 | 50 |
| A375 | >100 | | >200 | 100 | 10 | 40 |
| MCF-7 | >100 | | >100 | 100 | 50 | 60 |

[a]$LD_{50}$ was defined as the concentration of either TNF or caffeine necessary to achieve 50% cytotoxicity. The $LD_{50}$ values were obtained from a linear representation of dose-response curves of Figure 1 using the least squares method.

**Figure 2** Effects of TNF on DNA synthesis and cell division of L929 cells. Exponentially growing L929 cells were cultured in medium containing 20 μM of lovastatin for 30 h. Fresh medium supplemented with 2 mM mevalonic acid (control) or these reagents plus TNF at 10 ng $ml^{-1}$ (TNF-treated) was added at the time zero on the abscissa. At the indicated time intervals, [$^{3}$H]thymidine incorporation (lines) and cell number (circles) were measured. Each data point was performed in duplicate and the experiment was repeated at least three times with similar results.

enhancement observed in the cell lines WEHI, L929 and HeLa was TNF and caffeine dose-dependent and consistent with a synergistic interaction, as determined by an isobologram equation (data not shown).

### *Cell cycle specific effects of TNF and caffeine*

The mechanism involved in the interaction between TNF and caffeine on induction of cell death and its dependence on cell cycle events was further studied using L929 cells as a model. The action of TNF on the transition of cells throughout the cell cycle was examined by measuring the [$^{3}$H]thymidine incorporation into cellular DNA. As shown in Figure 2, DNA synthesis was slightly attenuated in cells growing in the presence of TNF. However, DNA synthesis in both control and TNF-treated cells reached a maximum at 15 h. Control cells reached the second S phase peak at 28 h, whereas DNA synthesis was inhibited in TNF-treated cells. Consistent with this finding, TNF-treated cultures failed to increase in number after 48 h, whereas the cell number of control cultures doubled (Figure 2).

Subsequent experiments were carried out to examine whether the duration of exposure to TNF was important in determining the synergistic interaction between TNF and caffeine. It was observed that the cytotoxicity was much less pronounced when L929 cells (Figure 3a) were exposed to TNF for short periods (4–12 h), as compared with the standard period of 24 h (Figure 1). Interestingly, the experiments also showed that cells became progressively insensitive to caffeine when the period of incubation was greater than 24 h (Figure 3b).

Next we examined whether the addition of TNF at advanced times along the $G_1$/S phase period could change the patterns of response of cells to caffeine. This possibility was examined by giving TNF at 4, 8 or 12 h following the release from lovastatin block. The results (Figure 3c) indicated that cells became less sensitive to the effects of TNF as they progressed throughout the cell cycle. Taken together, these findings suggest that L929 cells are much more sensitive to the effects of TNF during early $G_1$ phase (0–8 h). In contrast, cells treated with TNF during early $G_1$ phase are maximally susceptible to caffeine action during the time of transition from $G_2$ to M phase of the cell cycle (22–26 h).

### *Apoptosis is the predominant mode of cell death induced by TNF and caffeine in L929 cells*

Apoptosis is readily identified by staining cells with DNA-specific dyes (Duvall & Wyllie, 1986; Arends *et al.*, 1990; Gerschenson & Rotello, 1992). In this study we have scored apoptotic cells on the basis of the condensation state of chromatin and the uniform compaction of condensed masses along the inner border of the nuclear membrane (Figure 4). As shown in Table II, a significant number of apoptotic cells was identified among the population of cells treated with TNF alone for 30 h. The percentage of apoptotic cells increased from 27% to 53% and 67% when cells cultured with TNF for 24 h were exposed to caffeine (5 and 10 mM, respectively) for only 6 h (Table II). Apoptosis was further confirmed by comparing the integrity of isolated cellular DNA by 3′-end labelling and agarose gel electrophoresis. The results showed (Figure 5) that the characteristic DNA ladder pattern of apoptosis was induced by TNF in L929 cells and it was not present in the controls. Consistent with the morphological observations (Table II), the experiments also showed that the addition of caffeine at concentrations of 5 mM and 10 mM induced a greater extent of DNA fragmentation and a higher proportion of low molecular weight fragments than that observed in cells treated with TNF alone.

### Discussion

In this report we have shown that caffeine enhances the lethality of TNF when cells exposed to TNF for 24 h are then treated with various concentrations of caffeine.The synergistic interaction between these two agents on the induction of cell death was clearly observed in the cell lines WEHI, L929 and HeLa, but was less apparent in the cell lines A-375 and MCF-7 (Table I). The mechanism by which TNF sensitises cells to the effects of caffeine is still unclear. There have been a number of studies showing that caffeine and other methylxanthine analogues potentiate the lethal action of the chemical agents, thiotepa (Fingert *et al.*, 1986), nitrogen mustard (Lau & Pardee, 1982; Fingert *et al.*, 1986),

**Table II** Percentage of cell death via apoptosis in L929 cell cultures following TNF and caffeine treatment

| *Treatment* | *Concentration* | *Apoptotic cells (%)*[a] |
|---|---|---|
| None | | 0 |
| TNF (ng $ml^{-1}$) | 10 | 27.22 ± 1.2[b] |
| + Caffeine (mM) | 5 | 53.66 ± 1.4 |
| + Caffeine (mM) | 10 | 67.90 ± 2.7 |

L929 cells synchronised in $G_1$ phase were incubated in the absence or presence of TNF for 24 h. Caffeine was then added and the incubation continued for another 6 h. [a]Apoptotic cells were identified on the basis of morphological changes of their chromatin, including its condensation state and the uniform compaction of condensed masses at the border of nuclear membrane. [b]Results are from a representative experiment and are expressed as the mean percentage of apoptotic cells ± s.e.m. from ten fields evaluated.

**Figure 3** Effects of the time of exposure to TNF and the cell cycle position of the cells on the rates of enhancement of TNF lethality by caffeine in L929 cells. **a**, Cells synchronised at $G_1$ phase were incubated with various concentrations of TNF for shorter periods of time (as indicated) and then caffeine (20 mM) was added. After an additional 24 h of incubation the cell survival was determined. **b**, Cells synchronised at $G_1$ phase were incubated with various concentrations of TNF for the longer periods of time (as indicated) and then caffeine (20 mM) was added. After an additional 20–24 h of incubation the cell survival was determined. **c**, Cells synchronised at $G_1$ phase were incubated with various concentrations of TNF at the successive times of 0 h, 4 h, 8 h and 12 h following the initial time of incubation with fresh medium (time zero). Caffeine (20 mM) was given 24 h after TNF addition and the cells incubated for an additional 20–24 h. The percentage of survival was calculated by comparing the values obtained from control cells treated with caffeine (100%) to those from TNF plus caffeine-treated cells. Each point represents the means of at least two independent experiments. The s.d.s were less than 15% of the means.

**Figure 4** Morphological changes associated with apoposis induced by TNF and caffeine in L929 cells. **a**, shows the normal morphology of L929 cells from control cultures. **b**, shows the presence of typical apoptotic cells (arrows) as result of treatment of cells with TNF (10 ng $ml^{-1}$) for 24 h and caffeine (10 mM) for 6 h. In contrast to morphologically normal cells, the apoptotic cells have nuclei with either an extensive condensation of chromatin, several small condensed bodies at nuclear periphery or a reduced nuclear size.

hydroxyurea (Steinmann *et al*., 1991) as well as the physical agents, ultraviolet light (Rauth, 1967) and radiation (Busse *et al*., 1978). The common characteristic of these agents is their ability to induce DNA lesions that lead cells to arrest during S phase or $G_2$ phase of the cell cycle. Evidence within the literature suggests that the exposure of cells to TNF results in the generation of free radicals and oxidative DNA damage (Wong *et al*., 1989; Zimmerman *et al*., 1989) and growth arrest at the $G_2$ phase of the cell cycle (Darzynkiewicz *et al*., 1984). Therefore, it is possible that the DNA damage and perturbations in the cell cycle induced by TNF may be the factors that sensitise cells to the effects of caffeine.

Previous studies have demonstrated that the cytotoxic action of TNF (Darzynkiewicz *et al*., 1984; Coffman *et al*., 1989; Belizario & Dinarello, 1991) and caffeine (Lau & Pardee, 1982; Steinmann *et al*., 1991) is linked with the cell cycle. The results of experiments with L929 cells have also revealed that both the duration of exposure (Figure 3a,b) and the point in the cell cycle at which cells are exposed to TNF (Figure 3c) and caffeine (Figure 3a,b) appear to influence greatly the response of cells to these agents. The finding that cells in early $G_1$ phase are more susceptible to the effects of TNF, as compared to cells in late $G_1$ or early S phase (Figure 3c), appears to be related to the time necessary for full

**Figure 5** DNA fragmentation induced by TNF and caffeine. **a**, Agarose gel electrophoresis of 3′-end labelled total DNA extracts from L929 cells derived from cultures with no treatment (C, control) or with TNF at 10 ng ml$^{-1}$ in the absence (0 mM) or presence of 5 or 10 mM caffeine. Sizes of DNA fragments in basepairs (bp) were estimated following comparison of migration distance to a 123-bp DNA ladder. **b**, Quantitative analysis of the extent of internucleosomal DNA cleavage present in samples depicted in Panel **a** as assessed by β-counting of excised gel fractions containing low molecular weight (<15 kb) DNA fragments. The results are expressed as the mean ± s.d. of duplicate determinations from one representative experiment.

sensitisation of cells (DNA damage) and the arrival at $G_2$ phase (20–24 h). As noted before, the strongest effects of caffeine in a cell whose DNA is damaged would be expected during the arrest at $G_2$ phase following accumulation of critical levels of mitosis-inducing proteins, including $p34^{cdc2}$ protein kinase and cyclin B, that have been implicated in the mechanism of action of caffeine (Schlegel & Pardee, 1987; Steinmann *et al.*, 1991). Since the cell cycle length in human A-375 and MCF-7 cell lines is longer than 24 h (unpublished observation) and caffeine was added at this time, this may explain their reduced response (Table I), as compared to the mouse cell lines, WEHI and L929, and the human cell line, HeLa, in which the cell cycle length is less than 24 h (unpublished observation). It is important to mention, however, that these human cell lines are considerably more resistant to the lytic effects of TNF than the mouse cell lines, even if the cells are assayed using the standard method for TNF cytotoxicity which combines recombinant human TNF and actinomycin D (unpublished observation). Furthermore, the relationship between the poor response of certain cell lines to TNF and the number and/or type of TNF receptors (Heller *et al.*, 1992) has not been explored.

It is now clear that several mechanisms of cellular defense take place at different stages of the TNF cytotoxic pathway to protect cells from death. For example, expression of the enzyme manganous superoxide dismutase (MnSOD) evoked by IL-1, TNF (Wong & Goeddel, 1988) or gene transfection (Wong *et al.*, 1989) results in cellular resistance to TNF cytotoxicity. Consistent with these findings, it was observed here that after a longer period of incubation with TNF, L929 cells became gradually insensitive to caffeine (Figure 3b). Another mechanism known to affect cell survival is DNA repair capability. In this regard, it is interesting to note that murine cells have a reduced repair capacity compared to human cells (Bohr *et al.*, 1986). Furthermore, it is becoming clear that the control mechanisms to ensure the proper order of the cell cycle events (checkpoints) appear to be less stringent or relaxed in rodent compared to human cell lines (Kung *et al.*, 1990). More importantly, such differences profoundly affect their vulnerability to drug-induced mitotic aberrations and cytotoxicity (Kung *et al.*, 1990; Steinmann *et al.*, 1991). Thus, there are likely multiple, but not yet fully characterised, factors and mechanisms that influence the sensitivity of cells to TNF in a way that affects the action of caffeine.

TNF is capable of inducing both of the most well-characterised modes of cell death: necrosis and apoptosis (Laster *et al.*, 1988). Cells die via necrosis quite early after extensive injuries to the plasma membrane and cellular organelles. In apoptosis, cell death is slow and occurs long after DNA degradation, the condensation of chromatin and its marginalisation at the edges of the nuclear membrane (Duvall & Wyllie, 1986; Arends *et al.*, 1990). Although apoptosis is a common event seen during normal embryonic development and tissue renewal, it can also occur after major perturbations in vital cellular processes caused by cytotoxic chemical agents (Barry *et al.*, 1991; Sen & D'Incalci, 1992). The hallmark biochemical event in apoptosis is the cleavage of double-stranded DNA between nucleosomal units producing a typical ladder pattern of DNA fragmentation (Arends *et al.*, 1990). Because our results (Figure 4 and 5) are consistent with these criteria, we have concluded that apoptosis is the mode by which L929 cells, as well as WEHI and HeLa cells (unpublished observation), die in response to TNF. We have also obtained strong evidence suggesting that caffeine itself or its secondary mediator(s) act on molecules and/or reactions involved in this process to enhance the TNF lethality in L929 cells. Caffeine has diverse effects on many aspects of cell metabolism (Somani & Gupta, 1988). Of particular relevance in the current context is the reported ability of caffeine to increase intracellular levels of $Ca^{2+}$ and cAMP (Somani & Gupta, 1988), induce chromatin condensatin (Schlegel & Pardee, 1987), inhibit the DNA repair mechanism (Selby & Sancar, 1990), bind to adenosine receptors (Daly *et al.*, 1991) and activate $p34^{cdc2}$ protein kinase (Steinmann *et al.*, 1991). All of these mediators and biochemical events directly or indirectly participate in various stages seen in apoptosis (Gerschenson & Rotello, 1992; Trump & Berezesky, 1992; Kizaki *et al.*, 1990; Duvall & Wyllie, 1986; Arends *et al.*, 1990). Thus, we have postulated that caffeine may modulate or accelerate TNF cytotoxicity by altering the levels of cytosolic $Ca^{2+}$ and cAMP which may subsequently affect the activity of endonucleases, kinases or phosphatases required for apoptotic cell death.

Along with it cytotoxic effects in certain cancer cells, TNF plays a key role in the inflammatory process and immunity (Fiers, 1991). Among other activities, this cytokine activates phagocytosis, induces degranulation and superoxide ion production in polymorphonuclear cells, and increases endothelial cell permeability. These events lead to a series of pathophysiological situations which may be detrimental or even lethal to the host if the production of TNF persists for a long time (Fiers, 1991). It is of interest that caffeine and other methylxanthines reportedly suppress production of TNF (Dezube *et al.*, 1990) and various aspects of the TNF-mediated immune system cell functions (Zheng *et al.*, 1990; Dezube *et al.*, 1990; Fiers, 1991). Therefore, it could be predicted that methylxanthines, while enhancing the cytotoxicity of TNF, may also reduce its undesired side effects.

We thank Dr H. Fletcher Starnes Jr for his support during the early stages of this work; Drs Arthur B. Pardee, K. Keyomarsi and Andrew Kung for helpful discussions; Robert T. Schimke for providing lab and reagents for cytological analysis of apoptotic cells and A.W. Alberts of Merck Sharp and Dohme Research Laboratories for generous supply of Lovastatin. We also thank John Moriarity and David Tinsley for their technical assistance. J.E.B. was supported by a fellowship grant from the Ministry of Science and Technology of Brazil (CNPq) and J.L.T. by National Research Service Award HD 07556.

### References

AGARWAL, S., DRYSDALE, B.-E. & SHIN, H. (1988). Tumor necrosis factor-mediated cytotoxicity involves ADP-ribosylation. *J. Immunol.*, **140**, 4187–4192.

ARENDS, M.J., MORRIS, R.G. & WYLLIE, A.H. (1990). Apoptosis, the role of the endonuclease. *Amer. J. Path.*, **136**, 593–608.

BALOCH, Z., COHEN, S. & COFFMAN, F.D. (1990). Synergistic interactions between tumor necrosis factor and inhibitors of DNA topoisomerases I and II. *J. Immunol.*, **145**, 2908–2913.

BARRY, M.A., BEHNKE, C.A. & EASTMAN, A. (1991). Activation of programmed cell death (apoptosis) by cysplatin, other anticancer drugs, toxins and hyperthermia. *Bioch. Pharm.*, **40**, 2353–2362.

BELIZARIO, J.E. & DINARELLO, C.A. (1991). Interleukin 1$\beta$, interleukin-6, tumor necrosis factor-$\alpha$, and transforming growth factor $\beta$1 increase cell resistance to tumor necrosis factor cytotoxicity by growth arrest in the $G_1$ phase of the cell cycle. *Cancer Res.*, **51**, 2379–2385.

BOHR, V.A., OKUMOTO, D.S. & HANAWALT, P.C. (1986). Survival of UV-irradiated mammalian cells correlates with efficient DNA repair in an essential gene. *Proc. Natl Acad. Sci. USA*, **83**, 3830–3833.

BUSSE, P.M., BOSE, S.K., JONES, R.W. & TOLMACH, L.J. (1978). The action of caffeine on X-irradiated HeLa cells. *Rad. Res.*, **76**, 292–307.

COFFMAN, F.D., HAVILAND, D.L., GREEN, L.M. & WARE, L.M. (1989). Cytotoxicity by tumor necrosis factor is linked with the cell cycle but does not require DNA synthesis. *Growth Factors*, **1**, 357–362.

DALY, J.W., HIDE, J.W., MULLER, C.E. & SHAMIM, M. (1991). Caffeine analogs: structure-activity relationships at adenosine receptors. *Pharmacology*, **42**, 309–321.

DARZYNKIEWICZ, Z., WILLIAMSON, B., CARSWELL, E.A. & OLD, L. (1984). Cell cycle-specific effects of tumor necrosis factor. *Cancer Res.*, **44**, 83–90.

DEZUBE, B.J., EDER, J.P. & PARDEE, A.B. (1990). Phase I trial of escalating pentoxifylline dose with constant dose of thiotepa. *Cancer Res.*, **50**, 6806–6810.

DUVALL, E. & WYLLIE, A.H. (1986). Death and the cell. *Immunol. Today*, **7**, 115–118.

FIERS, W. (1991). Tumor necrosis factor, characterization at the molecular, cellular and *in vivo* level. *FEBS Lett.*, **285**, 199–212.

FINGERT, H.J., CHANG, J.D. & PARDEE, A.B. (1986). Cytotoxic, cell cycle, and chromosomal effects of methylxanthines in human tumor cells treated with alkylating agents. *Cancer Res.*, **46**, 2463–2467.

GERSCHENSON, L.E. & ROTELLO, R.J. (1992). Apoptosis: a different type of cell death. *FASEB J.*, **6**, 2450–2455.

HARTWELL, L.H. & WEINERT, A.W. (1989). Checkpoints: controls that ensure the order to cell cycle events. *Science*, **246**, 629–634.

HELLER, R.A., SONG, K., FAN, N. & CHANG, D.J. (1992). The p70 tumor necrosis factor receptor mediates cytotoxicity. *Cell*, **70**, 47–56.

JAKOBISIAK, M., BRUNO, S., SKIERSKI, J.S. & DARZYNKIEWICZ, Z. (1991). Cell cycle effects of lovastatin. *Proc. Natl Acad. Sci. USA*, **88**, 3628–3632.

KEYOMARSI, K., SANDOVAL, L., BAND, V. & PARDEE, A.B. (1991). Synchronization of tumor and normal cells from $G_1$ to multiple cell cycles by lovastatin. *Cancer Res.*, **51**, 3602–3609.

KIZAKI, H., SUZUKI, K., TADAKUMA, T. & ISHIMURA, Y. (1990). Adenosine receptor-mediated accumulation of cyclic AMP-induced T-lymphocyte death through internucleosomal DNA cleavage. *J. Biol. Chem.*, **265**, 5280–5284.

KUNG, A.L., SHERWOOD, S.W. & SCHIMKE, R.T. (1990). Cell line-specific differences in the control of cell cycle progression in the absence of mitosis. *Proc. Natl Acad. Sci. USA*, **87**, 9553.

LASTER, S.M., WOOD, J.C. & GOODING, L.R. (1988). Tumor necrosis factor can induce both apoptotic and necrotic forms of lysis. *J. Immunol.*, **141**, 2629–2634.

LAU, C.C. 7 PARDEE, A.B. (1982). Mechanism by which caffeine potentiates lethality of nitrogen mustard. *Proc. Natl Acad. Sci. USA*, **89**, 2942–2946.

PARDEE, A.B. (1989). $G_1$ events and regulation of cell proliferation. *Science*, **246**, 603–608.

RAUTH, A.M. (1967). Evidence for dark-reactivation of ultraviolet light in mouse L cells. *Radiat. Res.*, **31**, 121–138.

SCHLEGEL, R. & PARDEE, A.B. (1987). Periodic mitotic events induced in the absence of DNA replication. *Proc. Natl Acad. Sci. USA*, **84**, 9025–9029.

SELBY, C.P. & SANCAR, A. (1990). Molecular mechanisms of DNA repair inhibition by caffeine. *Proc. Natl Acad. Sci. USA*, **87**, 3522–3525.

SEN, S. & D'INCALCI, M. (1992). Apoptosis, biochemical events and relevance to cancer chemotherapy. *FEBS Lett.*, **307**, 122–127.

SOMANI, S.M. & GUPTA, P. (1988). Caffeine: a new look at an age-old drug. *Intl. J. Clin. Pharm. Ther. Toxic.*, **26**, 521–533.

STEINMANN, K.E., BELINSKY, G.S., LEE, D. & SCHLEGEL, R. (1991). Chemical induced premature mitosis: differential response in rodent and human cells and the relationship to cyclin B synthesis and $p34^{cdc2}$/cyclin B complex formation. *Proc. Natl Acad. Sci. USA*, **88**, 6843–6847.

SUFFYS, P., BEYAERT, R., VAN ROY, F. & FIERS, W. (1988). Involvement of a serine protease in tumour-necrosis-factor-mediated cytotoxicity. *Eur. J. Biochem.*, **178**, 257–265.

TILLY, J.L., KOWALSKI, K.I., JOHNSON, A.L. & HSUEH, A.J.W. (1991). Involvement of apoptosis in ovarian follicular atresia and postovulatory regression. *Endocrinology*, **129**, 2799–2801.

TILLY, J.L. & HSUEH, A.J.W. (1993). Microscale autoradiographic method for the qualitative and quantitative analysis of apoptotic DNA fragmentation. *J. Cell. Physiol.*, **154**, 519–526.

TRUMP, B.F. & BEREZESKY, I.K. (1992). The role of cytosolic $Ca^{2+}$ in cell injury, necrosis and apoptosis. *Curr. Op. Cell Biol.*, **4**, 227–232.

WONG, G.H.W. & GOEDDEL, D.V. (1988). Induction of manganous superioxide dismutase by tumor necrosis factor: possible protective mechanism. *Science*, **242**, 941–944.

WONG, G.H.W., ELWELL, J.H., OBERLEY, J.H. & GOEDDEL, D.V. (1989). Manganous superoxide dismutase is essential for cellular resistance to cytotoxicity of tumor necrosis factor. *Cell*, **58**, 923–931.

ZHENG, H., CROWLEY, J.J., CHAN, J.C., HOFFMANN, H., HATHERILL, J.R., ISHIZAKA, A. & RAFFIN, T.A. (1990). Attenuation of tumor necrosis factor-induced endothelial cell cytotoxicity and neutrophil chemiluminescence. *Am. Rev. Respir. Dis.*, **142**, 1073–1078.

ZIMMERMAN, R.J., CHAN, A. & LEADON, S.A. (1989). Oxidative damage in murine tumor cells treated *in vitro* by recombinant human tumor necrosis factor. *Cancer Res.*, **49**, 1644–1648.